# Prehospital recognition and antibiotics for 999 patients with sepsis: protocol for a feasibility study

**DOI:** 10.1186/s40814-018-0258-8

**Published:** 2018-03-12

**Authors:** Chris Moore, Jenna Bulger, Matt Morgan, Timothy Driscoll, Alison Porter, Saiful Islam, Mike Smyth, Gavin Perkins, Bernadette Sewell, Timothy Rainer, Prabath Nanayakkara, Chukwudi Okolie, Susan Allen, Greg Fegan, Jan Davies, Theresa Foster, Nick Francis, Fang Gao Smith, Gemma Ellis, Tracy Shanahan, Robin Howe, Helen Snooks

**Affiliations:** 1 Welsh Ambulance Services NHS Trust, Wales, UK; 2 ILS2, Swansea University Medical School, Swansea University, Singleton Campus, Wales, SA2 8PP UK; 3 Cardiff and Vale University Health Board, Wales, UK; 4 University of Warwick, England, UK; 5 VU University Medical Centre, Amsterdam, The Netherlands; 6 Patient Representative, C/O Swansea University, Swansea, UK; 7 East of England Ambulance Service NHS Trust, England, UK; 8 University of Birmingham, England, UK; 9 Public Health Wales, Wales, UK

**Keywords:** Paramedic, Sepsis, Antibiotics, Prehospital

## Abstract

**Background:**

Sepsis is a common condition which kills between 36,000 and 64,000 people every year in the UK. Early recognition and management of sepsis has been shown to reduce mortality and improve the health and well-being of people with sepsis. Paramedics frequently come into contact with patients with sepsis and are well placed to provide early diagnosis and treatment.

We aim to determine the feasibility of undertaking a fully powered randomised controlled trial (RCT) to test the clinical and cost-effectiveness of paramedics obtaining blood cultures from and administering IV antibiotics to patients with sepsis, so we can make a decision about whether to proceed to a fully powered randomised controlled trial, which will answer questions regarding safety and effectiveness for patients and benefit to the National Health Service (NHS).

**Methods/design:**

This is an individually randomised, two-arm feasibility study for a randomised controlled trial with a 1:1 ratio. Sixty paramedics will receive training to assist them to recognise sepsis using a screening tool, obtain blood cultures, and provide IV antibiotics. If sepsis is suspected, paramedics will randomly allocate patients to intervention or usual care using their next sequential individually issued scratch card. Patients will be followed up at 90 days using linked anonymised data to capture length of hospital admission and mortality. We will also collect self-reported health-related quality of life (using SF-12) at this time. We will interview ten patients by telephone and hold a focus group with paramedics, to find out what they think about the intervention.

**Discussion:**

At the end of this study, we will make a recommendation about whether a full randomised controlled trial of paramedics obtaining blood cultures and administering IV antibiotics for sepsis is warranted, and if so, we will develop a proposal for research funding in order to take the work forward.

**Trial registration:**

ISRCTN, ISRCTN36856873

**Electronic supplementary material:**

The online version of this article (10.1186/s40814-018-0258-8) contains supplementary material, which is available to authorized users.

## Background

Sepsis is caused by the body’s dysregulated immune response to an infection. It is a time-critical condition which can rapidly lead to multi-organ failure and death [1, 2]. Sepsis has a mortality rate as high as 35%, killing between 36,000 and 64,000 people in the UK every year [3]. More people in the UK die from sepsis than lung cancer or breast and bowel cancer combined [4]. There is evidence to suggest that early diagnosis of sepsis and early administration of intravenous (IV) antibiotics can reduce morbidity and mortality from sepsis [4–6]. The prehospital phase of emergency medical care provides the earliest opportunity for identification of sepsis and delivery of immediate life-saving treatment for patients. It is known that recognition of sepsis in the ambulance can speed up care in the emergency department (ED) and that these patients get the required diagnostics and treatment sooner [7, 8]. Emergency medical services (EMS) personnel may, therefore, play a major role in the identification, pre-alert, and initial management of sepsis. Traditionally, EMS training has focused on assessing and managing ‘barn door’ presentations such as chest pain and ST segment elevation myocardial infarction, stroke and transient ischaemic attack, and acute trauma. To date there has been little focus on the acute assessment and management of sepsis, though the National Early Warning Score (NEWS) system is now used by some EMS providers. Approximately 50% of patients with sepsis in the ED arrive by ambulance [9, 10], with an average prehospital care time of 45 min [11]. This suggests an important window of opportunity for early recognition and care of sepsis before hospital arrival. Despite the importance of this condition and the need for rapid emergency care, the evidence base to support the use of prehospital antibiotics is weak [12, 13], with currently no well-defined prehospital protocol for sepsis in the UK. EMS personnel already play a key role in providing early recognition, initial treatment, and rapid transport for patients with other time-critical conditions; expanding the evidence base on the prehospital management of sepsis is crucial to determining whether earlier identification and management in the prehospital setting could improve health outcomes and speed up care at the ED.

### Feasibility study aim

The aim of this study is to determine the feasibility of undertaking a fully powered randomised controlled trial (RCT) to test the clinical and cost-effectiveness of paramedics obtaining blood cultures from and administering IV antibiotics to patients with sepsis.

### Feasibility study objectives

1. Intervention development:

To work with clinicians, paramedics, pharmacists, and service users to develop a prehospital intervention for sepsis, comprising:

- A protocol for collection of blood cultures, administration of antibiotics, and handover
- Training for paramedics to deliver the agreed protocol

2. Intervention feasibility:

- To assess paramedic uptake and satisfaction with the training package
- To assess paramedic compliance with treatment protocol
- To determine safety and acceptability of the intervention to patients and paramedics

3. RCT feasibility:

- To develop and test trial recruitment, randomisation, and data collection processes
- To assess sample size requirements and attrition rates
- To determine availability of outcome data
- To clarify primary and secondary outcome measures

4. Full trial planning:

- To assess our findings against our progression criteria and, if met,
- Draft a full trial application to the HTA Research Programme

### Progression criteria

We will assess whether or not to proceed to a full multi-site RCT based on the following progression criteria, all of which should be met within reasonable limits (for example, if the progression criterion is within 5% of the target, we will review reasons for this and consider modifications to protocol; if within 10%, we will critically review reasons for this and assess whether major changes to protocol are likely to improve the issue; if more than 10%, we will not progress. The limits for each progression criterion will be stated in our statistical analysis plan; arbitrary limits of 5 and 10% have been suggested here for convenience).

### Intervention feasibility

1. *Compliance with protocol by paramedics*—no less than 80% of patients recognised as eligible patients by study paramedics are randomised
2. *Acceptability of intervention to patients*—mean patient satisfaction in intervention group is not less than 80% of patient satisfaction in the control group
3. *Safety*—number of patients who experience adverse events has a difference of less than 10% between trial arms
4. *Recognition of sepsis* (*success of training*)
  (a) At least 50% of patients with sepsis who are attended by study paramedics are recognised as eligible for the study.
  (b) At least 70% of randomised patients are diagnosed with sepsis in hospital.

### RCT feasibility

5. *Acceptability of RCT to paramedics*—at least 60% of eligible paramedics agree to take part in the study
6. *Acceptability of RCT to patients*—dissent to take part in the study is 30% or less
7. *Retrieval of outcomes*—follow-up data for primary outcome suitable for fully powered trial can be collected for 70% or more of patients
8. *Equipoise*—findings indicate that we remain in equipoise about the effectiveness of paramedic obtained blood cultures and prehospital antibiotics for sepsis.
9. *Recruitment*—recruitment target met to at least 80%

### Design

Individually randomised (1:1) feasibility trial. The unit of randomisation is the individual patient.

### Setting

The prehospital environment in the urban geographic area serving UHW, Cardiff. Paramedics based at all ambulance stations and fire stations in the Cardiff and Vale of Glamorgan localities of the Welsh Ambulance Services NHS Trust (WAST) will be invited to take part in PhRASe.

### Participants

#### Patient eligibility

**Fig. 1:**
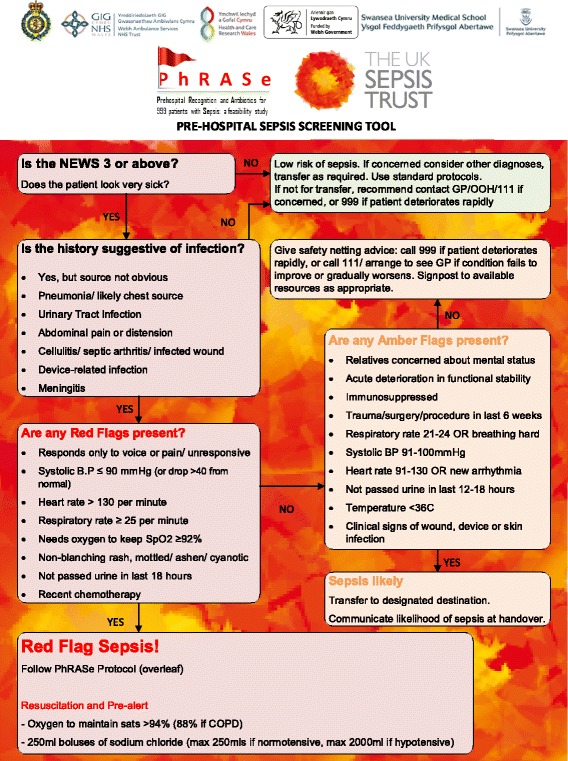
Screening tool

Inclusion criteria:

• Adult patients (18 years or older) with ‘Red Flag’ Sepsis (as defined by the Screening Tool seen in Fig. 1)

• Attended by study paramedics

• Will be conveyed to UHW

Exclusion criteria:

• Pregnancy (known or suspected)

• Known history of allergy to antibiotics

### Consent

It is acknowledged and accepted that it is not ethically appropriate to consent patients to research within the context of a medical emergency [14]. We have an experience of carrying out randomised trials in emergency care through the SAFER programme and TIER and RAPID feasibility trials [15, 16] and have successfully gained ethical, research, and information governance approvals to inform people of their inclusion in research following their attendance by emergency ambulance. At the time of paramedic attendance, therefore, patients will only be asked to provide consent to have blood cultures collected and receive antibiotics, should they be randomly allocated to the intervention arm. Verbal consent for these procedures will be taken by the paramedics, as would be normally taken for cannulation or venepuncture.

We will provide patients who have been recruited to the study with a Patient Information Sheet, which includes a ‘Participant Dissent Form’. This will be sent at 90 days (30 days for those recruited in the last two months of the study) after the patient’s 999 call. This gives the patient the option to:

1. Decline to receive any further correspondence from the study
2. Have all of their records withdrawn from the study

Any patients who dissent at this point are taken out of all further involvement, including anonymised follow-up. We have included service users in discussions about this approach which minimises intrusion and possible distress, whilst allowing the opportunity for informed involvement in patient follow-up through qualitative methods. The patient questionnaire sent at 90 days will include a question of whether the patient is willing to take part in an interview about their experiences of 999 care for sepsis. The Paramedic Research Support Officer (PRSO) will check the mortality status of patients before a Patient Information Sheet and questionnaire is sent to minimise distress to bereaved relatives. This means that patients who have deceased are included in our analysis as there is unfortunately no opportunity to give them the option to dissent. The Patient Information Sheet for the study can be seen in Additional file 1.

#### Sample size

Paramedics will recruit patients over a 6 month period. Based on data regarding throughput of sepsis cases in ED, we estimate a recruitment of approximately 100–150 patients. In this feasibility study, we will not be attempting to detect a clinically significant difference between the intervention and control groups in terms of outcomes, but rather to collect enough data to assess our methods against our progression criteria.

## Methods

### Usual care

At present, when a paramedic suspects a patient has sepsis, they will provide the patient with oxygen to maintain saturations over 94% (88% if the patient has chronic obstructive pulmonary disease) and give 250 ml boluses of 0.9% sodium chloride (up to 2000 ml if the patient is hypotensive, e.g. systolic blood pressure less than 100 mmHg, but clinical judgement can be used, taking into account the patient’s age and other clinical parameters). In addition, they will pre-alert the receiving hospital so that the patient can be taken directly to a resuscitation bay. During PhRASe, this care will continue to be given to both groups, with additional care given to patients in the intervention arm.

### Intervention development

A clinical intervention development group comprising paramedics, doctors, nurses, and pharmacists from prehospital care, emergency medicine, critical care, and microbiology met to define the exact treatment protocol and training methods for paramedics to follow early in the study period. They used clinical and local knowledge, research literature, and guidelines from the Joint Royal Colleges Ambulance Liaison Committee (JRCALC) and UHW. Based on advice from Public Health Wales, cefotaxime will be the antibiotic administered to patients in this feasibility study. The Screening Tool and Study Flowchart can be seen in Fig. 1; the screening tool is an adaptation of The UK Sepsis Trust’s Prehospital Sepsis Screening and Action Tool [17]. We decided only to include patients with Red Flag Sepsis in this feasibility study so that we did not broaden the use of antibiotics unnecessarily, in line with antimicrobial stewardship.

### Paramedic recruitment and training

We aim to recruit at least 60 of approximately 100 paramedics working in all ambulance stations and fire stations in the Cardiff and Vale of Glamorgan localities of WAST on a volunteer basis.

All study paramedics will be trained to recognise sepsis using the PhRASe screening tool, as well as to collect blood cultures and prepare and administer IV antibiotics. The training given to the paramedics will include the need to identify whether the patient has a known allergy to antibiotics, prior to administration, and the use of Aseptic Non Touch Technique (ANTT); although the collection of blood cultures within the hospital setting is routine, the collection of blood cultures in prehospital care is not a routinely practised skill for EMS staff and requires sound knowledge and practice of aseptic technique. The use of antibiotics (cefotaxime) will be supported by a patient group direction (PGD), which will allow the paramedics to administer a drug which is not routinely available to them under existing legal exemptions. Training will be delivered using formal group teaching sessions, e-learning, and one-to-one practical sessions.

### Randomisation

A randomisation schedule with a 1:1 ratio of ‘intervention’ or ‘control’ and stratified by paramedic will be produced by an independent statistician. Scratch cards will be produced to conceal the allocation, and a set of scratch cards will be issued to each study paramedic and be kept on their person in the pocket of their uniform during each working shift. When the study paramedic identifies an eligible patient, they will use their next sequential individually issued scratch card out of the sight of the patient. The unique number shown on the scratch card will become the patient’s study ID. This means the paramedic is always able to recruit a patient to the study and does not rely on having an Internet connection, a telephone signal, or a requirement to return to the ambulance to access the trial pack, to undertake the randomisation. The paramedic will retain the scratch card in order to store it with the randomisation log at the nurses’ station in UHW Resus, so that the PRSO can monitor randomisation.

### Blinding

Due to the nature of the intervention, the outcome of randomisation will not be blinded to the paramedics or patients. As data will be collected by the PRSO and handled by the data manager, they will also not be blinded to the allocation. The trial statistician will remain blinded to the allocation when conducting analysis.

### Outcomes

Part of the purpose of this study is to help us to define the primary outcome for a fully powered trial. Those to be tested include:

- Routinely collected anonymised data
- Mortality
- Length of hospital stay
- Patient reported (at 90 days)
- Health-related quality of life using SF-12

Secondary outcomes to be tested include:

1. Routinely collected anonymised data

- Length of intensive care unit stay
- Time from 999 call to administration of antibiotic by paramedic/in hospital
- Job cycle time, on-scene time
- Hospital diagnosis of sepsis or other condition
- Intervention cost
- Contamination of blood cultures

2. Patient reported (at 90 days)

- Patient satisfaction with care received by paramedics using quality of care monitor

### Data collection

#### Missed recruitments

There are two ways in which patients with sepsis who are attended by study paramedics may not be recruited:

1. The study paramedic does not recognise that the patient has sepsis
2. The study paramedic recognises that the patient has sepsis but does not randomise them

As part of this feasibility study, we will monitor for missed recruitments so that we can determine whether paramedics are able to accurately recognise patients with sepsis, whether paramedics are compliant with the protocol, and how many patients could not be randomised due to the exclusion criteria. The PRSO will compare recruited patients to all the patients admitted to UHW by study paramedics with sepsis. The PRSO will be able to discuss with individual paramedics any problems or concerns that they may have.

#### Quantitative

Data relating to clinical outcomes and hospital diagnosis will be obtained from the Secure Anonymised Information Linkage (SAIL) databank. The SF-12 and Quality of Care monitoring forms will be sent out to participants at 90 days (30 days for participants recruited in the last 2 months of the study) along with the Patient Dissent Forms to be returned directly to the research team at SU for data entry. Routine data will be used for paramedic call out information and will be collated by the PRSO. The PRSO will collect basic demographic details, i.e. age and gender, so that we can see whether participants’ baseline characteristics were similar between trial arms (indicating that randomisation was successful), as well as whether all patients who are allocated to intervention have both blood cultures taken and IV antibiotics given (as the paramedics may be unable to obtain blood cultures or give IV antibiotics in some cases). This information will be collected from patient’s WAST Patient Clinical Record.

#### Qualitative

Interview respondents will be sampled purposively [18] in a way previously used for Air Quality Alert scheme participants (findings not yet published). We will aim to explore with patients their experience of receiving the intervention and experience of recovery including overall quality of life. We will devise a semi-structured interview schedule, which will give the opportunity for patients to expand on information given on questionnaire forms about quality of life as well as asking for their views of the intervention through questions and prompts. We will sample 10 patients, selected purposively from those who receive IV antibiotics, to include a range of:

- Age
- Gender
- Time and day of attendance
- Study paramedic who assessed the patient

Interviews will be conducted over the telephone, as this is felt to be the most convenient and least intrusive to respondents.

Approximately ten paramedics will be invited to participate in focus group(s) to explore their views on the intervention. We will devise a topic guide consisting of a series of questions and prompts about whether they consider prehospital IV antibiotics an acceptable method of providing treatment for patients with sepsis compared with usual treatment and their experience of delivering the intervention. The focus group(s) will be conducted towards the end of patient recruitment and will be recorded and transcribed. We will also collect any complaints to WAST or CVUHB relating to the trial, should any be received.

All qualitative data will be recorded (with explicit permission to do so) and transcribed verbatim.

### Data handling

Study data will be collected and managed using REDCap (Research Electronic Data Capture) electronic data capture tools hosted at SU; the PRSO and project administrator will input the data into REDCap [19]. REDCap is a secure, web-based application designed to support data capture for research studies, providing (1) an intuitive interface for validated data entry, (2) audit trails for tracking data manipulation and export procedures, (3) automated export procedures for seamless data downloads to common statistical packages, and (4) procedures for importing data from external sources.

Data will be monitored on an on-going basis for completeness and precision by the data manager and through source data verification; it will be frozen as soon as possible thereafter to prevent changes made in error. All reasonable effort will be made to minimise missing data, and we shall adopt a consistent approach to missing data at the analysis stage (except where individual variables require otherwise).

Identifiable information will be seen only by the qualitative researcher in SU in order to contact them to invite them to take part in an interview (if the patient has stated they are happy to be contacted for this purpose). Qualitative data will be anonymously stored on password-protected computers at SU. Identifiable information will be removed from interview transcripts and stored separately and securely.

The PRSO will hold identifiable data securely only to enable us to collect anonymised outcomes for them via SAIL.

### Analysis

The analysis of quantitative data will mainly be descriptive. We will report baseline characteristics, the number of participants randomised to each arm, and the number who received the treatment allocated. Continuous outcomes (e.g. SF-12 Score; length of stay) will be analysed using *t* tests, or non-parametric equivalents, and we will report mean and standard deviation, or median and interquartile range, along with 95% confidence intervals. Categorical outcomes (e.g. mortality, presence of sepsis) will be analysed using chi-squared tests, and we will report raw counts, proportions (*n*, %), and 95% confidence intervals. We will conduct exploratory analysis only of our potential primary outcomes to determine whether we have met progression criterion 8 (whether we remain in equipoise), as well as to help to decide which is the most appropriate for use as the primary outcome in a fully powered trial. We will report against each of our progression criteria to determine whether we should seek further funding for a fully powered trial and provide the necessary estimates for performing the power calculation for the potential fully powered trial, if warranted. We will perform analysis using SPSS and report results using the relevant CONSORT checklists [20, 21].

We will use a thematic analysis approach for the qualitative data, analysing the patient and paramedic datasets separately, with input from at least two members of the research team to each analysis. We will look for consistency of viewpoint among respondents and will explore any deviations from the predominant view and possible reasons for this.

As this is a feasibility study, no formal health economic analysis will be undertaken. Health economics will focus on establishing the main cost drivers by estimating the intervention implementation cost, necessary parameters required (including measures used to collect costs/outcomes), and suitable framework to undertake a full cost-effectiveness analysis in a future trial. We will examine the feasibility of collecting data on outcomes and resource use by examining the completeness and response rate from the measures and consider solutions to collecting data in a full trial. We will investigate the design options of a future economic evaluation required including capturing longer-term horizons, e.g. any modelling that would be required alongside a future in-trial analysis of cost-effectiveness.

### Safety reporting

In the PhRASe feasibility study, safety reporting has two functions:

1. To monitor the safety of patients enrolled into the two arms of the trial;

2. To develop robust and practical safety reporting procedures for use in a fully powered multi-centred trial.

A principle of adverse event reporting is that safety should be monitored in both arms of the trial. Harms that occur after the patient has arrived at hospital could be a direct or indirect result of the treatment received in the prehospital environment. In some cases, we will be able to directly attribute the adverse event to the administration of antibiotics in the prehospital environment, but this will not be possible in all cases. We will monitor for the following serious adverse events (SAEs) in all patients up to 1 week:

• Anaphylaxis

• *C. difficile* infection

• Extravasation at the site administration of antibiotics

• Infection/cellulitis at the site of blood culture collection/administration of antibiotics

• Vascular damage at the site of blood culture collection/administration of antibiotics

SAE monitoring will be conducted by the PRSO so that the research team do not see any identifiable information. The PRSO will alert the Principal Investigator at UHW of all SAEs so that they can be assessed for seriousness and relatedness to the intervention. The Trial Manager will report any Suspected Unexpected Serious Adverse Reactions to the Research Ethics Committee, Sponsor, and the TSC’s Chair and Clinicians within 24 h of receiving notification of them. The rest of the TSC will be informed within 1 week, via the Chair.

We expect to capture harms beyond the 1-week follow-up period for SAEs through the outcomes for the trial, i.e. length of stay and mortality. Deaths and ICU admissions will be reported to the Trial Management Group on a three-monthly basis to ensure there are no safety concerns. For safety reporting purposes, this will be checked through routine records—this information will also be captured using the Secure Anonymised Information Linkage Databank, but due to a time lag in this information being available, we must also use alternative sources for interim reporting.

## Discussion

Service users have been involved in developing the initial research idea and preparing an application for funding for PhRASe, as well as in decision-making regarding all documentation for PhRASe, particularly the information sheet for patients. Service users will sit on the Trial Management Group and Trial Steering Committee throughout the trial to ensure that patient’s views are considered equally, as well as take part in analysis and dissemination of our findings.

Interviews and focus groups will be used as a source of information to develop the main trial (if warranted), for example, if suggestions are made as to how to improve the trial protocol.

It should be noted that PhRASe is not a Clinical Trial of Investigational Medicinal Product (CTIMP), as we are not testing the efficacy of antibiotics. Rather, we are testing the prehospital collection of blood cultures and administration of antibiotics in a technique which has not been previously tested by paramedics or in the prehospital setting.

We are aware that the nurses’ station in UHW resus, where the randomisation log will be kept during patient recruitment, is a very busy environment. We have taken steps to ensure that the log is not lost—including attaching it to the desk with string and placing a Bluetooth tracker on.

## Additional file

Additional file 1: Patient Information Sheet. (DOCX 696 kb)

## Abbreviations

CVUHB: Cardiff and Vale University Health Board
ED: Emergency department
EMS: Emergency medical services
IV: Intravenous
JRCALC: Joint Royal Colleges Ambulance Liaison Committee
NHS: National Health Service
PGD: Patient group direction
PRSO: Paramedic Research Support Officer
RCT: Randomised controlled trial
SAE: Serious adverse event
SU: Swansea University
UHW: University Hospital Wales
WAST: Welsh Ambulance Services NHS Trust
