# Supplementary material for: Prehospital recognition and antibiotics for 999 patients with sepsis: protocol for a feasibility study
Source: Pilot Feasibility Stud. 2018 Mar 12;4:64. doi: 10.1186/s40814-018-0258-8 (PMC5848520; doi:10.1186/s40814-018-0258-8)
Supplement: Supplementary file 1 — Patient Information Sheet. (DOCX 696 kb) [file 40814_2018_258_MOESM1_ESM.docx]

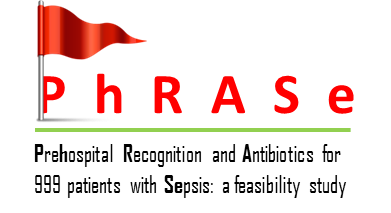


**Patient Information Sheet**

**Why am I being invited to take part?**

When you were very unwell recently, you received treatment from a paramedic. The paramedic who treated you identified you as someone who is eligible for our study which is exploring the potential role of paramedic administered antibiotics for sepsis. We would like to compare your experience with other patients who have been very unwell and would have received the same or different treatment to you. In this study, some patients with sepsis will receive antibiotics from paramedics and some will not.

The purpose of this information sheet is to tell you about the study, to help you decide whether you would wish to take part. Please take the time to read the information carefully and discuss it with friends or relatives if you wish. If you do not want to take part, any ongoing or future care you receive will not be affected in any way.


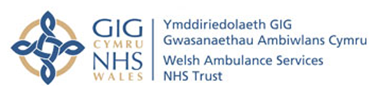
***Please ask the research team if there is anything which is unclear or if you would like more information.***

**What is the purpose of the PhRASe study?**

Sepsis is a serious condition, which unfortunately leads to thousands of deaths each year in the UK. If sepsis is recognised and managed early, patients are more likely to survive and have better health outcomes following their recovery. In this study paramedics will give antibiotics to some patients with sepsis before they are transported to hospital. We think that giving antibiotics before arriving at hospital may improve patient care, but this type of care is not normally given by paramedics, so we want to test whether it can be done safely and effectively. We aim to include 70 patients in this study and the results will help us decide if a larger study would be a good idea.

**Do I have to take part?**

No, taking part is entirely voluntary. You are free to withdraw at any time, without giving a reason.


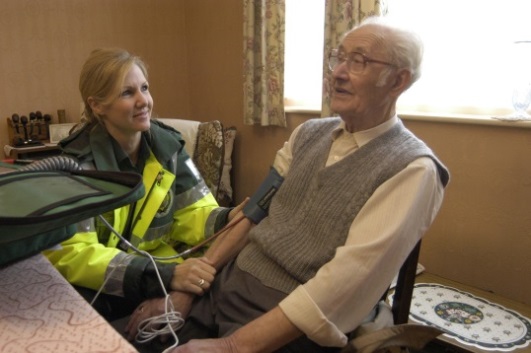


***You do not have to participate in all parts of the study but can choose to do some parts and not others.***

**What will happen if I do take part?**

| **If you agree to take**  **Part, we will:** | 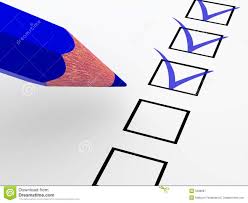 |
| --- | --- |

1. collect information about what happened to you when you were attended by an ambulance and about your hospital admission using anonymised linked data.

2. ask you to complete a questionnaire 90 days after your admission to hospital. The questions will ask how satisfied you were with the care you received from the paramedics who took you into hospital and about your quality of life. The questionnaire should take no more than fifteen minutes to complete.

3. ask a sample of 10 patients to take part in an interview with a researcher to help us understand the patients’ experience of their treatment.

We will ask you if you are willing to take part in an interview when we send your questionnaire.

**What are the benefits of taking part?**

By allowing us to follow up your ambulance and admission information, and by providing your answers, thoughts and feelings, you will help us to gain a better understanding of a patients experience when they suffer from sepsis. The information we obtain from this study may help to improve the treatment of people with sepsis in the future.

**What are the disadvantages of taking part?**

We realise we are asking you to give up your own time to complete a questionnaire, but have kept it as short as possible so that we do not inconvenience you for very long.

Taking part will not affect your ongoing or future care in any way. ****

**Will my participation be kept confidential in this study?**

Yes. We will follow ethical and legal practice to ensure all information about you will be handled in complete confidence. You will not be identifiable from any of the data shown to the research team. Your personal information (name, date of birth, home postcode and NHS number) will not be revealed in any audit, study report or publication at any time.

**What do I need to do now?**

If you are happy for your anonymised information to be used in this study, you do not need to do anything. You will receive a questionnaire to fill in approximately 90 days after you were admitted to hospital.

If you do not want to receive a questionnaire or further correspondence about this study, or if you do not want your anonymised information to be used in this study, please complete the form overleaf and return it to Swansea University in the enclosed pre-addressed envelope.

**Who should I contact for more information?**

| Chief Investigator: Chris Moore |
| --- |
| Tel: 07710152324  Email: [chris.moore@wales.nhs.uk](mailto:chris.moore@wales.nhs.uk) |
| Trial Manager: Jenna Bulger |
| Tel: 01792 513424  Email: [j.k.bulger@swansea.ac.uk](mailto:j.k.bulger@swansea.ac.uk) |

**If you have a concern about any aspect of this study, you should speak to one of the research team immediately. They will do their best to answer your questions (contact details listed above).**

For general advice about taking part in research, please see: http://www.nhs.uk/Conditions/Clinical-trials/Pages/Introduction.aspx

If you’re not happy with the care or treatment you have received, you have the right to complain. You can contact the Welsh Ambulance Services NHS Trust by email: [Amb_PuttingThingsRight@wales.nhs.uk](mailto:Amb_PuttingThingsRight@wales.nhs.uk), by letter: Tracy Myhill, Chief Executive, Welsh Ambulance Services NHS Trust, Trust Headquarters, HM Stanley Hospital, St Asaph, Denbighshire, LL17 0RS, or by telephone: 0300 321 3211.

**
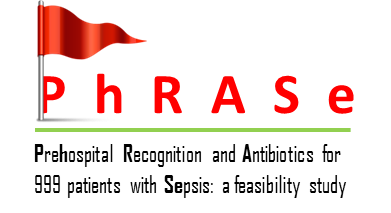
**

**PARTICIPANT DISSENT FORM**

| **Participant Study ID** |  |  |  |  |
| --- | --- | --- | --- | --- |

**Title of Project:**

Prehospital Recognition and Antibiotics for 999 patients with Sepsis (PhRASe): a feasibility study

**Research contacts:**

Mr Chris Moore (Welsh Ambulance Services NHS Trust - 07710152324) or

Dr Jenna Bulger (Swansea University - 01792 513424)

| 1. Please do not send any further correspondence |  |
| --- | --- |
| 1. Please withdraw all my records from this study |  |

| **Your name in capitals** | **Date** | **Signature** |
| --- | --- | --- |
|  |  |  |
